# Editorial: Fractal and Multifractal Facets in the Structure and Dynamics of Physiological Systems and Applications to Homeostatic Control, Disease Diagnosis and Integrated Cyber-Physical Platforms

**DOI:** 10.3389/fphys.2020.00447

**Published:** 2020-05-13

**Authors:** Paul Bogdan, András Eke, Plamen Ch. Ivanov

**Affiliations:** ^1^Ming-Hsieh Department of Electrical and Computer Engineering, University of Southern California, Los Angeles, CA, United States; ^2^Department of Physiology, School of Medicine, Semmelweis University, Budapest, Hungary; ^3^Department of Radiology and Biomedical Imaging, Yale University School of Medicine, New Haven, CT, United States; ^4^Keck Laboratory for Network Physiology, Department of Physics, Boston University, Boston, MA, United States; ^5^Harvard Medical School and Division of Sleep Medicine, Brigham and Women's Hospital, Boston, MA, United States; ^6^Institute of Solid State Physics, Bulgarian Academy of Sciences, Sofia, Bulgaria

**Keywords:** multifractals, physiological systems, control, disease diagnosis and analysis, cyber-physical systems (CPS)

A fundamental problem in biology, physiology, and medicine is understanding how complexity in the structure and dynamics of biological and physiological systems emerges from multicomponent regulatory mechanisms, where non-linear feedback loops across scales lead to efficient homeostatic control in the presence of continuous temporal variability in systems outputs. Addressing this problem requires (i) comprehensive analyses of systems dynamics based on multifractal formalism and methodology (Ivanov et al., 1999, 2001, 2002; Mukli et al., 2015) to probe feedback interactions underlying biological and physiological systems by quantifying the temporal organization of physiological fluctuations and their cascades across scales, and (ii) a general network physiology framework (Bashan et al., 2012; Ivanov and Bartsch, 2014; Bartsch et al., 2015; Ivanov et al., 2016) to investigate networks of interactions among diverse physiological systems and subsystems across space and time scales that lead to emergent complex behaviors at the organism level. An entire new class of diagnostic and prognostic biomarkers has resulted from pioneering studies in these new directions, especially needed now when witnessing a pandemic of chronic diseases (e.g., heart diseases, diabetes, and its complications, stroke, cancer, brain diseases) which constitute a significant cause of rising healthcare costs and a reduced quality-of-life (QoL).

Despite the increased need for smart healthcare sensing systems that monitor patients' body balance, there is no coherent theory that facilitates the modeling of human physiological processes and the design and optimization of future healthcare cyber-physical systems (HCPSs) (Bogdan and Marculescu, 2011; Xue and Bogdan, 2017; Bogdan, 2019). The HCPSs are expected to measure and mine the patient's physiological state based on available continuous sensing, quantify risk indices corresponding to the onset of abnormality, signal the need for critical medical intervention in real-time by communicating patient's medical information via a network from individual to hospital, and most importantly control (actuate) vital health signals (e.g., cardiac pacing, insulin level, blood pressure) within personalized homeostasis.

To maintain good health, prevent health complications, and/or avoid fatal conditions calls for a cross-disciplinary approach to HCPS design that rely on recent advances in statistical physics, non-linear dynamics, machine learning, and artificial intelligence. There is a palpable need for a multi-disciplinary approach to consolidate the current state of art in order to respond to the following fundamental challenges. First and foremost, the fundamental properties (e.g., non-stationarity, fractality) of human physiology in terms of mathematical formalism needs to be characterized in order to facilitate the understanding of its complexity (West, 1991; Bassingthwaighte et al., 1994; Stanley et al., 1999; Amaral et al., 2001; Eke et al., 2002; Ivanov et al., 2009; Xue and Bogdan, 2019) in healthy homeostasis, as well as in conditions associated with aging and diseases (Mietus et al., 2000; Ashkenazy et al., 2001; Bernaola-Galvan et al., 2001; Schulte-Frohlinde et al., 2001; Goldberger et al., 2002; Schmitt and Ivanov, 2007).

Indeed, defining the trajectory of the healthy aging process in terms of its complexity metrics (Goldberger et al., 2002) seems essential to keep the health care system on target. At this end, Mukli et al. demonstrated the utility of multifractal metrics of cerebral hemodynamics as biomarkers of the healthy aging process. In particular, these authors—by applying a novel adaptive bimodal multifractal analysis (Mukli et al., 2015; Nagy et al., 2017) to enhanced human cerebrocortical functional Near Infrared Spectroscopy (fNIRS) data—disentangled the neurogenic from vasogenic components in brain dynamics that were then shown being attenuated in the course of healthy aging. Racz et al. applied multifractal time series analysis (Mukli et al., 2015) to investigate dynamic functional connectivity (DFC) reconstructed from resting-state electroencephalography (EEG) measurements. This work demonstrated that metrics of DFC as captured in the temporal evolution of graph theoretical measures—even under resting-state conditions—dynamically fluctuated according to region-specific true multifractal temporal patterns. Mono- and multifractal measures of the generalized Hurst exponent for individual functional connections exhibited a spatial pattern well in sink with the overall functional organization of the brain. The authors propose that multifractal analysis can provide further details in the description of DFC to most methods currently applied in the field, and could serve as a valuable tool for a better characterization of healthy and pathological brain functions. Akhrif et al. performed an adaptive monofractal analysis of functional magnetic resonance (fMRI) data and estimated the Hurst exponent of the impulsivity network. This study not only demonstrates that the Hurst exponent can be used as a biomarker to quantify deviations in network functions at early stages, but also serve as a control knob in therapeutic strategies aimed at delaying the onset and improving treatment of disorders. França et al. demonstrated that multifractal analysis can provide important relevant information for mining the intracranially recorded EEG data and extracting features that can be used for machine learning-based diagnosis outperforming other techniques like signal variance or power spectrum. In particular, they demonstrated that there may exist an optimal time scale between the sampling frequency and epoch length that can best influence the detection accuracy of temporal changes in multifractality associated with epileptic seizures. While this study has identified that multifractal algorithms perform well on EEG and simulated data alike, it also brought attention to the issue of optimal time scales at which machine learning-based diagnosis should be done.

With the goal of mathematical characterization of blood glucose variability, initially scrutinized in Ghorbani and Bogdan (2013). Kohnert et al. provided a cross-sectional investigation and compared the relationships between indices of non-linear dynamics and traditional glycemic variability, as well as their potential application in diabetes control. Although this analysis showed that the Poincaré plot measures the multiscale entropy (MSE) index, and the detrended fluctuation analysis exponents can help to discriminate between the type 1 and type 2 diabetes (e.g., the MSE index decreased consistently from the non-diabetic to the type 1 diabetic group), it also highlighted the need to develop more advanced complexity measures in order to better characterize the glycemia. These fractal-based observations can have a significant impact on the development of efficacious artificial pancreas with increased patient's QoL.

In order to extract the multifractal characteristics and determine disease signatures, Reyes-Manzano et al. investigated the multifractal behavior of the beat-to-beat heart dynamics captured in RR-interval fluctuations in fibromyalgia patients (FM) via the multifractal detrended fluctuation analysis (Kantelhardt et al., 2002). The multifractal and non-linear behavior exhibited a decrease in patients with fibromyalgia. Consequently, this investigation not only highlights the role of the dysfunctional autonomic control in the pathogenesis of fibromyalgia, but it can also provide a theoretical and algorithmic foundation for HCPS. With the goal of analyzing the cardiac abnormalities observed in heart failure disease, Platiša et al. exploited the short-term and long-term scaling exponents obtained via the detrended fluctuation analysis (DFA) (Peng et al., 1995; Hu et al., 2001; Chen et al., 2002, 2005; Xu et al., 2005; Ma et al., 2010) for discriminating the deterioration in cardiac autonomic nervous system control. Their study demonstrated that the heart failure patients exhibited a more pronounced heart rate asymmetry and a higher long-term scaling exponent. Moreover, a ratio between the DFA short-term and long-term scaling exponents can help at discriminating between various types of heart failure disease states.

To provide a deeper understanding of atrial fibrillation disease, Attuel et al. described a model of cardiac excitable cell network which is capable to reproduce the experimentally observed multifractal intermittent nature of the cardiac impulse energy. In order to investigate the cardiac electrophysiological and arrhythmogenic properties, Tse et al. studied the beat-to-beat variability in action potential duration data and concluded that the atrial monophasic action potential recordings (MAPs) exhibits greater degree of variability than the ventricular MAPs. Along the same lines of exploiting non-linear metrics in various disease states, Ghita et al. investigated the tissue heterogeneity and dynamic non-linearity in respiratory impedance data and quantified the sensitivity of the forced oscillation technique to various degrees of obstruction in patients suffering from chronic obstructive pulmonary disease (COPD). They showed that the degree of non-linearity correlates well with various degrees of COPD.

We need rigorous mathematical techniques and a general theoretical framework to characterize the interactions between integrated physiologic systems with different output dynamics (Bartsch et al., 2012; Liu et al., 2015; Lin et al., 2016; Ivanov et al., 2017), as well as other related processes (e.g., metabolic, proteomic, genomic), and understand their role within the overall network physiology of healthy dynamics (Ivanov et al., 2016). Along the lines of characterizing the interactions across scales, Ghorbani et al. investigated the individual gene expression dynamics and the cross-dependency among genes and transcription factors in the context of gene regulatory networks corresponding to *Escherichia coli* and *Saccharomyces cerevisiae* bacteria. This initial study demonstrated that the interaction between genes and linked transcription factors exhibit multifractal and long-range cross-correlated characteristics with implications for understanding genome-level dynamics.

Finally, there is a fundamental need for deeper understanding of the mechanisms of stochastic feedback and variability in biological systems and physiological processes (Ivanov et al., 1998; Ashkenazy et al., 2002; Lo et al., 2002). This is essential for developing adequate approaches to mathematically characterize homeostasis as well as for defining new control strategies accounting for intra- and inter-patient specificity—a truly mathematical approach to personalized medicine (Xue and Bogdan, 2017; Bogdan, 2019; Yang and Bogdan, 2020). For example, the multi-scale interactions and feedback among cognitive events may play an essential role in information processing in the prefrontal cortex (Racz et al., 2017). Hu et al. demonstrated that the optimal performance of the working memory is concurrent with the critical dynamics at the network level and the excitatory and inhibitory balance at the neuron level. Moreover, this study suggests the existence of a unified multi-scale optimal state for the prefrontal cortex, which further can be modulated by dopamine opening new therapeutic strategies in HCPS.

The works presented in this Research Topic collection as well as current advances in the field of fractal and multi-fractal investigations of physiological systems structure and dynamics, and their applications to homeostatic control, clinical diagnosis, and the development of cyber-physical systems in healthcare outline a new horizon of multidisciplinary cooperation with new challenges. There is an urgent need for adopting a cross-scale perspective and a corresponding theoretical framework to investigate the multi-scale regulatory mechanisms underlying the overall network physiology and its relation to physiological states and functions emerging at the organism level in health and disease. When dealing with the heterogeneity, multi-modality and complexity of physiological processes, we need rigorous mathematical and algorithmic techniques that can extract causal interdependencies between systems across different scales while overcoming various noise sources. For example, obtaining high-frequency genomic and proteomic sensing data over large spatial and temporal dimensions can open new frontiers and lead to the discovery of basic laws of regulation with broad clinical applications. Consequently, progress in this direction will require new algorithmic strategies to quantify time-varying information flow among diverse physiological processes across scales, and determine how it influences the global dynamics of complex physiological networks. Intrinsically related with future efforts on quantifying causal dependencies and control principles in biological and physiological networks, it will be essential to develop robust optimization algorithms capable to reconstruct or infer the structure and dynamics of complex interdependent networks while overcoming partial observability, noise induced defects and adversarial interventions caused by bacterial or viral infections. Lastly, the biomedical and engineering communities need to develop new control methodologies that do not seek to only enforce a specific reference value (that proved beneficial for some patients), but rather ensure that the physiological complexity and multifractality are restored to the healthy profiles when abnormalities are detected—e.g., a mathematical strategy to abstract the complexity of brain network through an approximate transfer function and a new minimal control strategy allows one to efficiently enforce a healthy fractal profile when frailty is early detected.

Toward this end, with these challenges also unique opportunities arise for interdisciplinary research. From the interactions of statistical physics, non-linear dynamics, information theory, probability and stochastic processes, artificial intelligence, machine learning, control theory and optimization, basic physiology and medicine new theoretical and algorithmic foundations will emerge for mining, analyzing, and controlling the network physiology. Ultimately, such efforts would lead to a new class of network-physiology-derived diagnostic and prognostic markers with innovative applications in cyber-physical systems and clinical practice.

## Author Contributions

All authors listed have made a substantial, direct and intellectual contribution to the work, and approved it for publication.

## Conflict of Interest

The authors declare that the research was conducted in the absence of any commercial or financial relationships that could be construed as a potential conflict of interest.

## References

[B1] Amaral L. A. N. Ivanov P. Ch. Aoyagi N. Hidaka I. Tomono S. Goldberger A. L.. (2001). Behavioral-independent features of complex heartbeat dynamics. Phys. Rev. Lett. 86:6026. 10.1103/PhysRevLett.86.6026 11415420

[B2] Ashkenazy Y. Hausdorff J. M. Ivanov P. Ch. Stanley H. E. (2002). A stochastic model of human gait dynamics. Physica A 316, 662–670. 10.1016/S0378-4371(02)01453-X

[B3] Ashkenazy Y. Ivanov P. Ch. Havlin S. Peng C.-K. Goldberger A. L. Stanley H. E. (2001). Magnitude and sign correlations in heartbeat fluctuations. Phys. Rev. Lett. 86, 1900–1903. 10.1103/PhysRevLett.86.1900 11290277

[B4] Bartsch R. P. Liu K. K. Bashan A. Ivanov P. Ch. (2015). Network physiology: how organ dynamically interact. PLoS ONE 10:e0142143. 10.1371/journal.pone.0142143 26555073 PMC4640580

[B5] Bartsch R. P. Schumann A. Y. Kantelhardt J. W. Penzel T. Ivanov P. Ch. (2012). Phase transitions in physiologic coupling. Proc. Natl. Acad. Sci. U.S.A. 109, 10181–10186. 10.1073/pnas.1204568109 22691492 PMC3387128

[B6] Bashan A. Bartsch R. P. Kantelhardt J. W. Havlin S. Ivanov P. Ch. (2012). Network physiology reveals relations between network topology and physiologic function. Nat. Commun. 3:702. 10.1038/ncomms1705 22426223 PMC3518900

[B7] Bassingthwaighte J. B. Liebovitch L. S. West B. J. (1994). Fractal Physiology. Oxford: American Physiological Society.

[B8] Bernaola-Galvan P. Ivanov P. Ch. Amaral L. A. N. Stanley H. E. (2001). Scale invariance in the nonstationarity of human heart rate. Phys. Rev. Lett. 87:168105. 10.1103/PhysRevLett.87.168105 11690251

[B9] Bogdan P. (2019). Taming the unknown unknowns in complex systems: challenges and opportunities for modeling, analysis and control of complex (Biological) collectives. Front. Physiol. 10:1452. 10.3389/fphys.2019.01452 31849703 PMC6903773

[B10] Bogdan P. Marculescu R. (2011). Towards a science of cyber-physical systems design, in Proceedings of the 2011 IEEE/ACM Second International Conference on Cyber-Physical Systems (ICCPS) (Chicago, IL), 99–108.

[B11] Chen Z. Hu K. Carpena P. Bernaola-Galvan P. Stanley H. E. Ivanov P. Ch. (2005). Effect of nonlinear filters on detrended fluctuation analysis. Phys. Rev. E 71:011104. 10.1103/PhysRevE.71.011104 15697577

[B12] Chen Z. Ivanov P. Ch. Hu K. Stanley H. E. (2002). Effect of nonstationarities on detrended fluctuation analysis. Phys. Rev. E 65:041107. 10.1103/PhysRevE.65.041107 12005806

[B13] Eke A. Herman P. Kocsis L. Kozak L. R. (2002). Fractal characterization of complexity in temporal physiological signals. Physiol. Meas. 23, R1–R38. 10.1088/0967-3334/23/1/201 11876246

[B14] Ghorbani M. Bogdan P. (2013). A cyber-physical system approach to artificial pancreas design, in Proceedings of the Ninth IEEE/ACM/IFIP International Conference on Hardware/Software Codesign and System Synthesis (Montreal, QC: CODES+ISSS), 1–10.

[B15] Goldberger A. L. Amaral L. A. N. Hausdorff J. M. Ivanov P. Ch. Peng C.-K. H. Eugene Stanley H. E. (2002). Fractal dynamics in physiology: alterations with disease and aging. Proc. Natl. Acad. Sci. U.S.A. 99, 2466–2472. 10.1073/pnas.012579499 11875196 PMC128562

[B16] Hu K. Ivanov P. Ch. Chen Z. Carpena P. Stanley H. E. (2001). Effect of trends on detrended fluctuation analysis. Phys. Rev. E 64:011114. 10.1103/PhysRevE.64.011114 11461232

[B17] Ivanov P. Ch. Amaral L. A. N. Goldberger A. L. Havlin S. Rosenblum M. G. Stanley H. E.. (2001). From 1/f noise to multifractal cascades in heartbeat dynamics. Chaos 11, 641–652. 10.1063/1.1395631 12779503

[B18] Ivanov P. Ch. Amaral L. A. N. Goldberger A. L. Havlin S. Rosenblum M. G. Struzik Z. R.. (1999). Multifractality in human heartbeat dynamics. Nature 399, 461–465. 10.1038/20924 10365957

[B19] Ivanov P. Ch. Amaral L. A. N. Goldberger A. L. Stanley H. E. (1998). Stochastic feedback and the regulation of biological rhythms. Europhys. Lett. 43, 363–368. 10.1209/epl/i1998-00366-3 11542723

[B20] Ivanov P. Ch. Bartsch R. P. (2014). Network physiology: mapping interactions between networks of physiologic networks, in Networks of Networks: The Last Frontier of Complexity. Understanding Complex Systems, eds D'Agostino G. Scala A. (Cham: Springer). 10.1007/978-3-319-03518-5_10

[B21] Ivanov P. Ch. Goldberger A. L. Stanley H. E. (2002). Fractal and multifractal approaches in physiology, in The Science of Disasters: Climate Disruptions, Heart Attacks and Market Crashes, eds Bunde A. Kropp J. Schellnhuber H.-J. H-J (Berlin: Springer Verlag), 219–257.

[B22] Ivanov P. Ch. Liu K. K. L. Bartsch R. P. (2016). Focus on the emerging new fields of network physiology and network medicine. New J. Phys. 18:100201. 10.1088/1367-2630/18/10/100201 30881198 PMC6415921

[B23] Ivanov P. Ch. Liu K. K. L. Lin A. Bartsch R. P. (2017). Network physiology: from neural plasticity to organ network interactions, in Emergent Complexity from Nonlinearity, in Physics, Engineering and the Life Sciences. Springer Proceedings in Physics, Vol. 191 eds Mantica G. Stoop R. Stramaglia S. (Cham: Springer), 145–165.

[B24] Ivanov P. Ch. Ma Q. D. Y. Bartsch R. P. Hausdorff J. M. Amaral L. A. N. Schulte-Frohlinde V.. (2009). Levels of complexity in scale-invariant neural signals. Phys. Rev. E 79:041920. 10.1103/PhysRevE.79.041920 19518269 PMC6653582

[B25] Kantelhardt J. W. Zschiegner S. A. Koscielny-Bunde E. Havlin S. Bunde A. Stanley H. E. (2002). Multifractal detrended fluctuation analysis of nonstationary time series. *Phys. A Stat. Mech*. Appl. 316, 87–114. 10.1016/S0378-4371(02)01383-3

[B26] Lin A. Liu K. K. L. Bartsch R. P. Ivanov P. Ch. (2016). Delay-correlation landscape reveals characteristic time delays of brain rhythms and heart interactions. Philos. Trans. R. Soc. A 374:20150182. 10.1098/rsta.2015.0182 27044991 PMC4822443

[B27] Liu K. K. L. Bartsch R. P. Ma Q. D. Y. Ivanov P. Ch. (2015). Major component analysis of dynamic networks of physiologic organ interactions. J. Phys. Conf. Ser. 640:012013. 10.1088/1742-6596/640/1/012013 30174717 PMC6119077

[B28] Lo C.-C. Amaral L. A. N. Havlin S. Ivanov P. Ch. Penzel T. Peter J.-H. (2002). Dynamics of sleep-wake transitions during sleep. Europhys. Lett. 57, 625–631. 10.1209/epl/i2002-00508-7

[B29] Ma Q. D. Y. Bartsch R. P. Bernaola-Galvan P. Yoneyama M. Ivanov P. Ch. (2010). Effects of extreme data loss on long-range correlated and anticorrelated signals quantified by detrended fluctuation analysis. Phys. Rev. E 81:031101 10.1103/PhysRevE.81.031101 PMC3534784 20365691

[B30] Mietus J. E. Peng C. K. Ivanov P. Ch. Goldberger A. L. (2000). Detection of obstructive sleep apnea from cardiac interbeat interval time series, in Computers in Cardiology, Vol.27 (Cat. 00CH37163) (Cambridge, MA), 753–756.

[B31] Mukli P. Nagy Z. Eke A. (2015). Multifractal formalism by enforcing the universal behavior of scaling functions. Phys. A Stat. Mech. Appl. 417, 150–167. 10.1016/j.physa.2014.09.002

[B32] Nagy Z. Mukli P. Herman P. Eke A. (2017). Decomposing multifractal crossovers. Front. Physiol. 8:533. 10.3389/fphys.2017.00533 28798694 PMC5527813

[B33] Peng C.-K. Havlin S. Stanley H. E. Goldberger A. L. (1995). Quantification of scaling exponents and crossover phenomena in nonstationary heartbeat time series. Chaos 5, 82–87. 10.1063/1.166141 11538314

[B34] Racz F. S. Mukli P. Nagy Z. Eke A. (2017). Increased prefrontal cortex connectivity during cognitive challenge assessed by fNIRS imaging. Biomed. Opt. Express 8, 3842–3855. 10.1364/BOE.8.003842 28856054 PMC5560845

[B35] Schmitt D. T. Ivanov P. Ch. (2007). Fractal scale-invariant and nonlinear properties of cardiac dynamics remain stable with advanced age: a new mechanistic picture of cardiac control in healthy elderly. Am. J. Physiol. Regul. Integr. Comp. Physiol. 293, R1923–R1937. 10.1152/ajpregu.00372.2007 17670859

[B36] Schulte-Frohlinde V. Ashkenazy Y. Ivanov P. Ch. Glass L. Goldberger A. L. Stanley H. E. (2001). Noise effects on the complex patterns of abnormal heartbeats. Phys. Rev. Lett. 87:068104. 10.1103/PhysRevLett.87.068104 11497867

[B37] Stanley H. E. Amaral L. A. N. Goldberger A. L. Havlin S. Ivanov P. Ch. Peng C. K. (1999). Statistical physics and physiology: monofractal and multifractal approaches. Phys. A Stat. Mech. Appl. 270, 309–324. 10.1016/S0378-4371(99)00230-7 11543220

[B38] West B. (1991). Fractal Physiology and Chaos in Medicine. Singapore: World Scientific Pub. Co. Inc.

[B39] Xu L. Ivanov P. Ch. Hu K. Chen Z. Carbone A. Stanley H. E. (2005). Quantifying signals with power-law correlations: a comparative study of detrended fluctuation analysis and detrended moving average techniques. Phys. Rev. E 71:051101. 10.1103/PhysRevE.71.051101 16089515

[B40] Xue Y. Bogdan P. (2017). Constructing compact causal mathematical models for complex dynamics, in Proceedings of 8th ACM/IEEE International Conference on Cyber-Physical System (ICCPS), Cyber-Physical Systems Week (Pittsburgh, PA).

[B41] Xue Y. Bogdan P. (2019). Reconstructing missing complex networks against adversarial interventions. Nat. Commun. 10:1738. 10.1038/s41467-019-09774-x 30988308 PMC6465316

[B42] Yang R. Bogdan P. (2020). Controlling the multifractal generating measures of complex networks. Nat. Sci. Rep. 10:5541. 10.1038/s41598-020-62380-6 32218468 PMC7098978

